# Type I interferon subtypes differentially activate the anti-leukaemic function of natural killer cells

**DOI:** 10.3389/fimmu.2022.1050718

**Published:** 2022-11-24

**Authors:** Samantha A. Barnes, Katherine M. Audsley, Hannah V. Newnes, Sonia Fernandez, Emma de Jong, Jason Waithman, Bree Foley

**Affiliations:** ^1^ Telethon Kids Institute, The University of Western Australia, Nedlands, WA, Australia; ^2^ School of Biomedical Sciences, The University of Western Australia, Crawley, WA, Australia

**Keywords:** natural killer cells, leukemia, interferon subtypes, adoptive cell therapy, immunotherapy

## Abstract

Natural killer (NK) cells have an intrinsic ability to detect and eliminate leukaemic cells. Cellular therapies using cytokine-activated NK cells have emerged as promising treatments for patients with advanced leukaemia. However, not all patients respond to current NK cell therapies, and thus improvements in efficacy are required. Type I interferons (IFN-I) are a family of potent immunomodulatory cytokines with a known ability to modulate NK cell responses against cancer. Although the human IFN-I family comprises 16 distinct subtypes, only IFNα2 has been widely explored as an anti-cancer agent. Here, we investigated the individual immunomodulatory effects each IFNα subtype and IFNβ had on NK cell functionality to determine whether a particular subtype confers enhanced effector activity against leukaemia. Importantly, IFNα14 and IFNβ were identified as superior activators of NK cell effector function *in vitro*. To test the ability of these subtypes to enhance NK cell activity *in vivo*, IFN-I stimulation was overlaid onto a standard *ex vivo* expansion protocol to generate NK cells for adoptive cell therapy. Interestingly, infusion of NK cells pre-activated with IFNα14, but not IFNβ, significantly prolonged survival in a preclinical model of leukaemia compared to NK cells expanded without IFN-I. Collectively, these results highlight the diverse immunomodulatory potencies of individual IFN-I subtypes and support further investigation into the use of IFNα14 to favourably modulate NK cells against leukaemia.

## Introduction

Natural killer (NK) cells are a cytotoxic group of innate lymphoid cells that play an important role in the elimination of virally infected and malignant cells. Over the past two decades the therapeutic antitumour potential of NK cells has become well established across the settings of allogeneic haematopoietic stem cell transplantation and adoptive cell therapy (ACT) [reviewed (1)]. Following repeated success in clinical trials, donor-derived NK cell therapies have emerged as a safe and effective treatment option for patients with haematological malignancies such as leukaemia. While consistent progress has been made over the past 20 years, not all patients respond to current NK cell therapies. As such, the development and optimisation of strategies which can potently enhance NK cell anti-leukaemic function has become a major focus of the field.

Type I interferons (IFN-I) are well-known for their capacity to modulate NK cell immunity (2). IFN-I has been shown to be critical in controlling NK cell anti-tumour responses in several murine models [reviewed (2)] including limiting metastasis formation in a breast cancer model (3). Additionally, IFN-I has also been shown to play an important role in NK cell development (4), homeostasis (5), and the formation of memory responses (6). Despite their role in immunity often being regarded universal, 16 functional IFN-I subtypes exist. These include 12 distinct IFNα subtypes (IFNα1, -α2, -α4, -α5, -α6, -α7, -α8, -α10, -α14, -α16, -α17 and -α21), IFNβ, and the lesser-known IFNϵ, IFNκ, and IFNw (7). Although each subtype binds to the common IFNα/β receptor (IFNAR), their biological activities are not equivalent. In a viral setting individual IFN-I subtypes harbour differing capacities to suppress viral replication (8, 9) and to modulate CD4^+^ and CD8^+^ T cell responses (10, 11) and NK cell activity (11, 12). Additionally, we have recently demonstrated that individual IFN-I subtypes also harbor differing capacities for driving antitumor immunity in murine models of melanoma (13, 14); however, this has yet to be explored in a human setting. To date, IFNα2 (IFNα2B) remains the only IFNα subtype clinically approved for the treatment of cancer (15).

While earlier studies have explored the ability of a small subset of IFNα subtypes to augment NK cell cytotoxicity *in vitro* (16), there has been no systematic analysis to determine whether a particular IFN-I subtype confers enhanced NK cell responses against leukaemia. As such, it remains possible that several of the untested IFNα subtypes or IFNβ may drive a more potent NK cell response than the clinically approved IFNα2B subtype. Here, we aim to close this knowledge gap by investigating the individual immunomodulatory properties of the 12 IFNα subtypes and IFNβ on NK cells. We directly compared the effect each IFN-I subtype has on enhancing NK cell functionality, focussing on their ability to promote NK cell degranulation, cytokine production and polyfunctionality against target cells *in vitro*, upregulate expression of activation markers and cytotoxic molecules, activate STAT signalling pathways, and synergise with an established *ex vivo* pre-activation strategy to generate therapeutic NK cells for ACT.

In a cohort of 50 healthy adult donors, we report that nearly all IFN-I subtypes tested were more effective at enhancing NK cell degranulation and cytokine production in response to target cell stimulation than the clinical IFNα2B subtype. From this initial screen we identified three top candidate subtypes (IFNα6, IFNα14, IFNβ) with increased ability to upregulate activation markers and cytotoxic effector molecules, enhanced potential for JAK/STAT signalling, and improved capacities to prime NK cells to respond to respond to IL-15-mediated signalling. Furthermore, when incorporated into a standard *ex vivo* expansion protocol to generate NK cells for ACT, these subtypes were superior at increasing NK cell anti-leukaemic activity over IFNα2B. Importantly, adoptive transfer of IFNα14-activated NK cells significantly prolonged survival in a preclinical model of leukaemia compared to control NK cells expanded without IFN-I. These results demonstrate the need to take into consideration the different potential of IFN-I subtypes to enhance NK cell anti-leukaemic responses and support further clinical development of these subtypes for optimal cancer treatments.

## Materials and methods

### NK cell donors

Blood was obtained from 50 blood donors attending The Australian Red Cross Blood Service, Western Australia, with informed consent obtained in accordance with the Declaration of Helsinki. Written approval to use blood samples was obtained from the University of Western Australia (RA/4/1/7311). Peripheral blood mononuclear cells (PBMCs) were purified by density centrifugation using Lymphoprep (Stemcell Technologies) and cryopreserved. Before analysis, the thawed cells were incubated overnight at 37°C in complete media (R10) (RPMI (Life Technologies) supplemented with 10% foetal calf serum (CellSera), 100 U mL^-1^ penicillin, 100 mg mL^-1^ streptomycin, 2 mM GlutaMax, 50 mM 2-ME, 1% sodium pyruvate and 1% non-essential amino acids (all Life Technologies)). Where indicated, cells were immediately stimulated following thawing with either 100 IU mL^-1^ IFNα (PBL Assay Science) or IFNβ (Stemcell Technologies) for 16-18 hours prior to analysis.

### K562 cell line

The MHC class I-negative cell line K562 was obtained from the American Type Culture Collection (ATCC). Cells were maintained in complete media (R10) and used within three weeks after thawing.

### Functional flow assay

Expression of CD107a and production of TNFα and IFNγ were measured as described previously (17). Briefly, PBMCs were either left unstimulated or were stimulated overnight. The next day, cells were washed and then co-incubated with K562 target cells at an effector-to-target (E:T) ratio of 2:1 for 5 hours. Anti-CD107a (H4A3) was added at the start of culture. Brefeldin A and monensin (both BD Biosciences) were added after 1 hour. The following antibodies were used: anti-CD56 (clone B159), anti-CD3 (SK7), anti-IFNγ (B27), anti-TNFα (MAb11) and fixable viability stain FV575 (all BD Biosciences). Cells were analysed on an BD LSR Fortessa X-20 and using FlowJo Version 10 software (BD Biosciences).

### Phenotyping of NK cells

NK cell expression of activation markers and cytotoxic effector molecules was assessed using the following antibodies: anti-CD56 (B159), anti-CD3 (SK7), anti-NKG2D (1D11), anti-NKp30 (p30-15), anti-NKp46 (9E2/NKp46), anti-CD2 (RPA-2.10), anti-CD108 (KS-2), anti-CD160 (BY55), anti-CD244 (C1.7), anti-granzyme B (GB11) and anti-perforin (δG9). Cells were analysed on an BD LSR Fortessa X-20 and using FlowJo Version 10 software.

### Phosflow

PBMCs were either left unstimulated or were stimulated with 100 IU mL^-1^ IFNα or IFNβ overnight (pSTAT5) or for 30 min (pSTAT1 and pSTAT3). For pSTAT5 analysis, cells were washed and re-stimulated with 5 ng mL^-1^ IL-15 for 15 min. Cells were then immediately fixed in Lyse/Fix Buffer and permeabilised in Perm Buffer III (both BD Biosciences). The following antibodies were used: anti-CD56 (B159), anti-CD3 (SK7), anti-pSTAT1 (pY701), anti-pSTAT3 (pY705) and anti-pSTAT5 (pY694) (all BD Biosciences). Cells were analysed on a BD LSR Fortessa X-20 and using FlowJo version 10 software.

### NK cell expansion

PBMCs were thawed on day -1 and cultured at 3x10^6^ cells mL^-1^ in R10. The following day, NK cells were expanded with irradiated K562 cells at a 6:1 ratio in R10 supplemented with 10 IU mL^-1^ (increasing to 100 IU mL^-1^ on day 7) recombinant human IL-2 (Life Technologies) and 5 ng mL^-1^ recombinant human IL-15 (Peprotech), replacing half the media every 2-3 days and splitting 1:2 (<4x10^6^ cells mL^-1^) or 1:4 (≥4x10^6^ cells mL^-1^) on day 7. Where indicated, 100 IU mL^-1^ IFNα or IFNβ was added on day -1 and/or day 13. NK cells were collected on day 14, washed, and effector function was assessed. For *in vivo* experiments, NK cells were expanded as described above in CellGenix^®^ GMP Stem Cell Growth Medium (Sartorius CellGenix), either with or without addition of 100 IU mL^-1^ IFNα14 or IFNβ on day -1 and day 13. Cells were collected on day 14 and washed three times in PBS in preparation for adoptive transfer.

### Adoptive NK cell transfer

Eight- to ten-week-old NOD.Cg-Prkdc^scid^ Il2rg^tm1Wjl^/SzJ (NSG) mice were purchased from the Animal Research Centre, Perth, Australia. Animals were housed under specific pathogen-free conditions and all studies were approved by the Animal Ethics Committee, Telethon Kids Institute, Perth, Australia. Mice received adoptive transfer of 1x10^6^ K562 cells on day 0. Following engraftment of leukaemic cells mice received adoptive transfer of 4.5x10^6^ NK cells on day 4. Mice received thrice weekly intraperitoneal injections of 0.5 mg recombinant human IL-15 (Miltenyi Biotech) starting on the day of adoptive NK cell transfer and continuing for the following two weeks. Mice were then monitored for disease progression and were euthanised once symptoms of leukaemia developed.

### Statistics

Data were summarized with mean and standard error of the mean (mean ± SEM). Where appropriate, data were tested for normal distribution using the Shapiro-Wilk normality test. For comparisons between independent samples, the Student’s t-test was used. For comparisons of matched samples, the paired t-test was used. For *in vivo* experiments, length of survival between groups was compared using the Log Rank (Mantel-Cox) test. Statistical significance was indicated as *p < 0.05, **p < 0.01, ***p < 0.001 and ****p < 0.0001. Statistical analyses were performed using Prism 8 (GraphPad Software).

## Results

### Individual IFN-I subtypes display different capacities to modulate NK cell effector function against leukaemia *in vitro*

**Figure 1:**
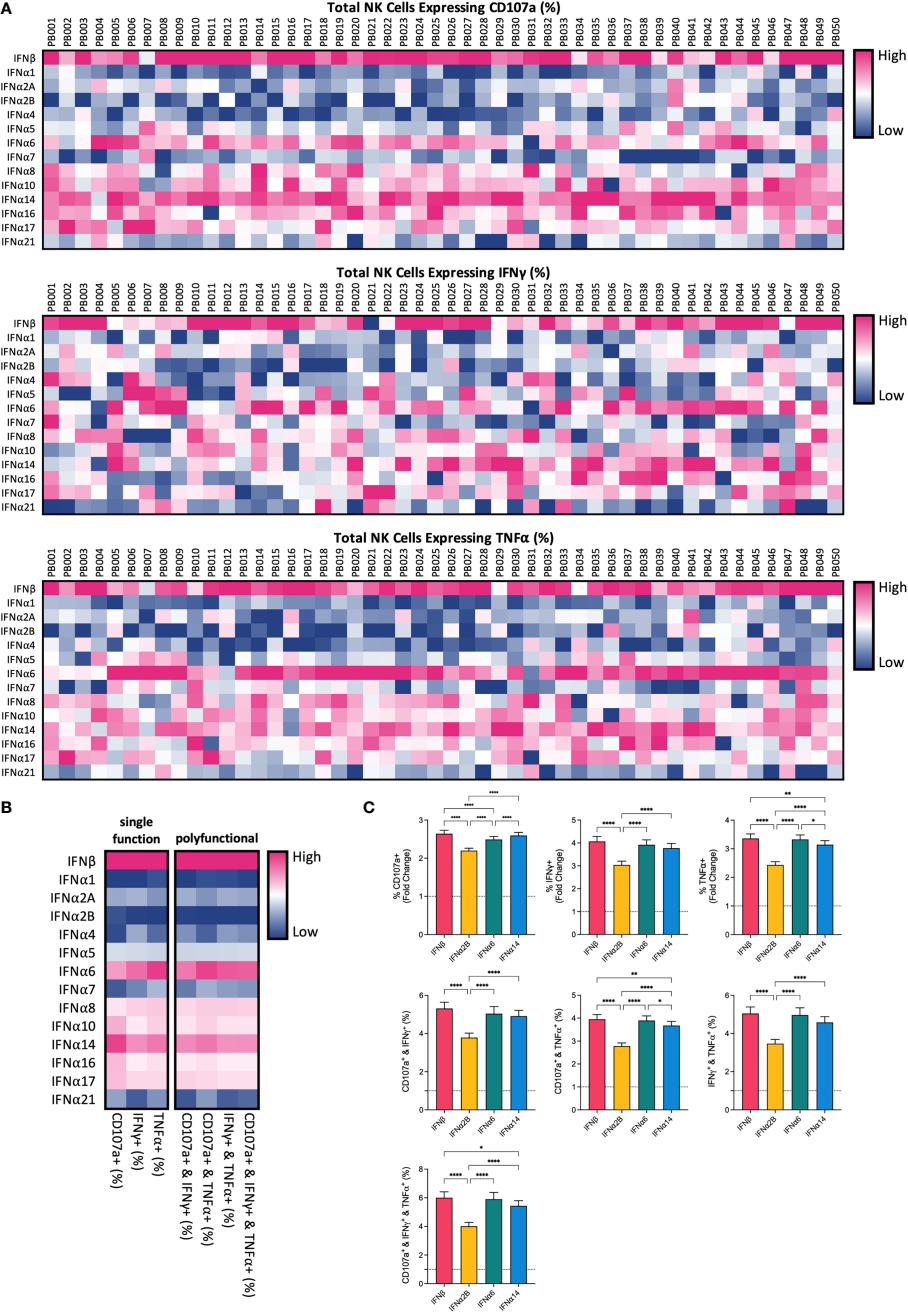
IFN-I subtypes differentially activate NK cell effector functions PBMCs from 50 healthy donors (PB001-PB050) were either left unstimulated (baseline; data not shown) or were stimulated with 100 IU mL^-1^ of each IFNα; subtype or IFNβ overnight. Following overnight activation, cells were washed and then co-incubated with K562 cells for 5 hours at a 2:1 E:T ratio and NK cell effector functions were assessed. **(A)** Heatmaps summarizing the percentage of CD56^+^CD3^neg^ NK cells expressing CD107a (range: 17.40-81.10%) or producing IFNy (range: 4.19-50.20%) or TNFα (range: 2.24-74.4%) for each donor in response to individual IFN-I subtypes. **(B)** The mean percentage of NK cells expressing CD107a, IFNy or TNFα, or a combination of two or more of these functional markers, was calculated for each IFN-I subtype across all donors. **(C)** Summary data show mean ± SEM fold change difference in the percentage of NK cells expressing CD107a, IFNy or TNFα, or a combination of two or more of these functional markers, following stimulation with selected IFN-I subtypes compared to baseline (dotted line). Data were compared using paired Student’s *t* tests. *p < 0.05, **p < 0.01, ****p < 0.0001.

To compare the potential of each individual IFN-I subtype to activate NK cells, we carried out a comprehensive *in vitro* screening assay of NK cell anti-leukaemic activity. PBMCs from 50 healthy donors were left unstimulated (baseline) or were stimulated with or 100 IU mL^-1^ of each IFNα subtype or IFNβ overnight (16-18 hours). Cells were then washed and co-cultured with the class-I negative leukaemic cell line K562 for 5 hours, after which NK cells were assessed for their capacity to degranulate (as evidenced by expression of CD107a on the cell surface) or produce cytokines (IFNγ and TNFα) (**Supplementary Figure 1A**). Stimulation with IFN-I enhanced NK cell degranulation and production of IFNγ and TNFα compared to baseline (24.83% ± 1.19%, 7.6% ± 0.64% and 13.68% ± 1.18%, respectively; data not shown), with individual IFN-I subtypes modulating NK cell effector functions to varying extents (**Figure 1A**). Despite variability between individual donors, the pattern of enhanced NK cell activity was largely maintained across the donor pool for each IFN-I subtype. Stimulation with IFNβ, IFNα6 or IFNα14 resulted in the most robust increase in NK cell degranulation, cytokine production and polyfunctionality on average across all donors (**Figure 1B**; **Supplementary Figure 1B**). IFNβ and IFNα14 stimulation resulted in the highest mean percentage of total NK cells expressing CD107a (61.04% ± 1.27% and 60.34% ± 1.35%, respectively), whereas IFNβ and IFNα6 stimulation resulted in the highest levels of IFNγ (26.74% ± 1.4% and 25.23% ± 1.26%, respectively) and TNFα production (39.86% ± 1.96% and 39.26% ± 1.92%, respectively). IFNβ stimulation also promoted the greatest levels of polyfunctionality, resulting in the highest mean percentage of NK cells co-expressing CD107a and IFNγ (24.27% ± 1.35%), CD107a and TNFα (37.54% ± 1.95%), IFNγ and TNFα (21.89% ± 1.37%), and CD107a and IFNγ and TNFα (20.66% ± 1.33%). Furthermore, stimulation with IFNβ, IFNα6 or IFNα14 resulted in a significantly greater fold-change increase in NK cell degranulation, cytokine production and polyfunctionality compared to baseline than the clinically approved subtype IFNα2B (**Figure 1C**). As such, we selected these top three candidate subtypes (IFNα6, IFNα14 and IFNβ) to carry forward in this study, alongside the representative clinical subtype IFNα2B.

### IFN-I stimulation primes NK cells for cytotoxicity

**Figure 2:**
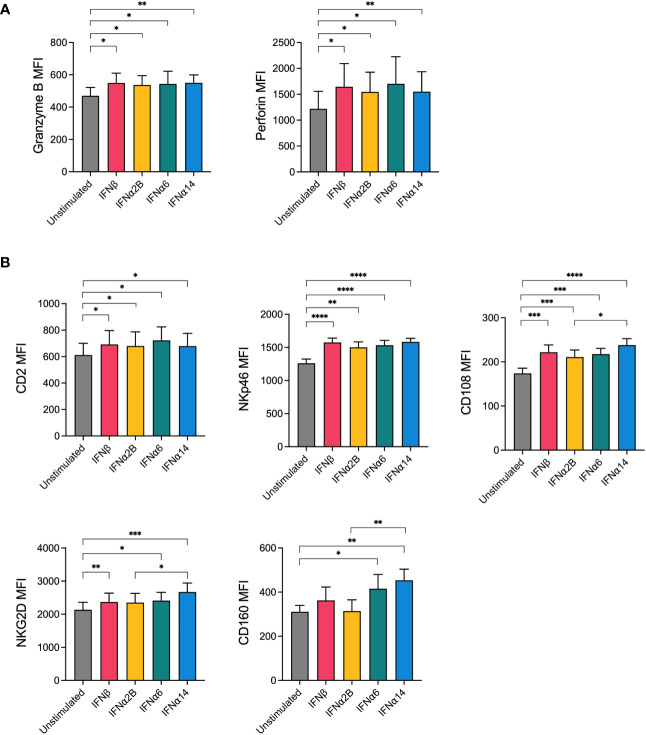
NK cells display increased expression of cytotoxic effector molecules and activation markers following IFN-I activation PBMCs were either left unstimulated or were stimulated with 100 IU mL^-1^ of IFNβ, IFNα2B, IFNα6 or IFNα14 overnight. Following overnight activation, CD56^+^CD3^neg^ NK cells were assessed for the expression of granzyme B and perforin or for the expression of five activation markers (CD2, NKp46, CD108, NKG2D, CD160). **(A)** Summary plots show the mean ± SEM granzyme B or perforin MFI in unstimulated and IFN-I stimulated NK cells. **(B)** Summary plots show the mean ± SEM MFI of the indicated activation markers in unstimulated and IFN-I stimulated NK cells. n = 8-13 healthy donors. Data were compared using paired Student’s *t* tests. *p < 0.05, **p < 0.01, ***p < 0.001, ****p < 0.0001.

The release of granules containing cytotoxic proteins, such as perforin and granzyme B, is the major mechanism through which NK cells lyse target cells (18). Since activation with IFNα6, IFNα14 and IFNβ significantly enhanced NK cell degranulation in response to leukaemic target cells, we next evaluated the effect of IFN-I stimulation on the expression of these cytotoxic proteins. Overnight stimulation with each of the selected IFN-I subtypes (IFNβ, IFNα2B, IFNα6 or IFNα14) was found to significantly increase expression of granzyme B and perforin compared to baseline (unstimulated) (**Figure 2A**). However, no significant differences in expression levels were identified between the IFN-I subtypes.

NK cell activation relies on the complex and hierarchical integration of signals through various activating receptors (19). To investigate how our top candidate IFN-I subtypes prime NK cells for enhanced effector activity, we next sought to investigate the effect of IFN-I stimulation on the expression of activating and costimulatory NK cell receptors. Following overnight stimulation with either media alone (unstimulated) or each of the selected IFN-I subtypes, NK cells were analysed for expression of several key markers involved in NK cell activation, adhesion and functional potential including NKG2D, NKp30, NKp46, CD244 (2B4), CD2, CD108 and CD160. No difference in the expression of 2B4 or NKp30 was observed following IFN-I stimulation (data not shown). Stimulation with each of the selected IFN-I subtypes resulted in an increase in the expression of CD2, NKp46, CD108 and NKG2D compared to baseline, whereas CD160 expression was selectively increased by stimulation with IFNα6 and IFNα14 but not IFNβ or IFNα2B (**Figure 2B**). Interestingly, IFNα14 stimulation also resulted in significantly greater expression of CD108, NKG2D and CD160 compared to IFNα2B.

### Selected IFN-I subtypes differentially activate STAT1 and STAT3 signalling pathways

**Figure 3:**
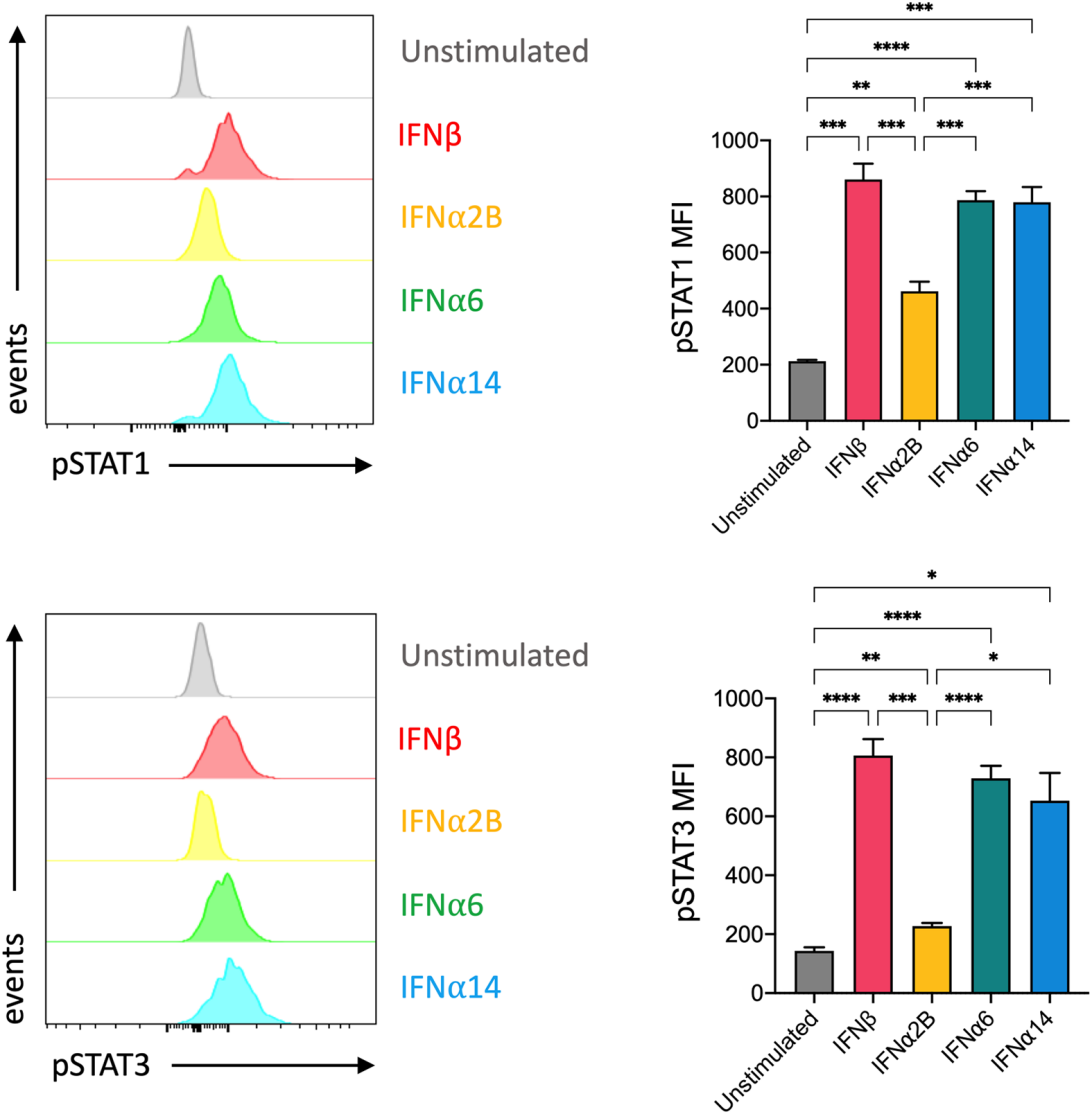
IFNβ, IFNα6 and IFNα14 activation induces stronger phosphorylation of STAT1 and STAT3 in NK cells than the clinical standard IFNα2B PBMCs were assessed for the expression of phosphorylated STAT1 (pY701) or STAT3 (pY705) at baseline (unstimulated) and following stimulation with 100 IU mL^-1^ of IFNβ, IFNα2B, IFNα6 or IFNα14 for 30 mins. Representative histogram plots show per-cell pSTAT1 or pSTAT3 expression in CD56^+^CD3^-^ NK cells. Summary data show mean ± SEM pSTAT1 or pSTAT3 MFI in unstimulated and IFN-I stimulated NK cells. n = 7 healthy donors, 2 independent experiments. Data were compared using a paired Student’s *t* test. *p < 0.05, **p < 0.01, ***p < 0.001, ****p < 0.0001.

Despite all binding to the common IFNAR, it has been reported that individual IFN-I subtypes drive distinct downstream signalling cascades, and thus biological effects, through phosphorylation of various STAT molecules (20). In NK cells, IFN-I stimulation predominantly activates STAT1 and, to a lesser degree, STAT3 signalling (21). STAT1 and STAT3 are major regulators of NK cell cytotoxicity and IFNγ production (22–25). As such, we investigated the individual capacity for each of our top-performing IFN-I subtypes to activate STAT1 and STAT3 signalling in NK cells. We observed a significant increase in the expression of phosphorylated STAT1 (pSTAT1) and phosphorylated STAT3 (pSTAT3) following stimulation with each of the selected IFN-I subtypes compared to baseline (**Figure 3**). Strikingly, stimulation with IFNβ, IFNα6 and IFNα14 also resulted in significantly higher expression of both pSTAT1 and pSTAT3 compared to IFNα2B.

### IFNα6 and IFNα14 stimulation primes NK cells to respond to IL-15

**Figure 4:**
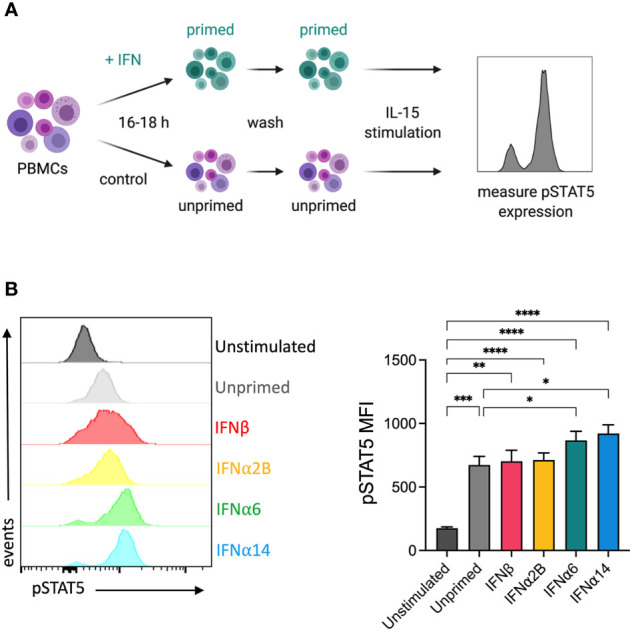
IFNα6 and IFNα14 activation primes NK cells to respond to IL-15 stimulation **(A)** Experimental design. PBMCs were either left unstimulated or were primed with 100 IU mL^-1^ of IFNβ, IFNα2B, IFNα6 or IFNα14 overnight. Following overnight activation, cells were washed and then assessed for the expression of phosphorylated STAT5 (Y694) at baseline (unstimulated) or following stimulation with 5 ng mL^-1^ IL-15 for 15 mins. **(B)** Representative histogram plot shows per-cell pSTAT5 expression in unstimulated, unprimed, and IFN-I primed CD56^+^CD3^-^ NK cells. Summary data show mean ± SEM pSTAT5 MFI in unstimulated, unprimed, and IFN-I primed NK cells. n = 10 healthy donors, 3 independent experiments. Data were compared using a paired Student’s *t* test. *p < 0.05, **p < 0.01, ***p < 0.001, ****p < 0.0001.

It has previously been reported that IFNα stimulation primes NK cells to respond to IL-15, increasing IL-15-mediated phosphorylation of STAT5 and subsequently enhancing cytotoxicity against K562 target cells (26). To study the capacity of individual IFN-I subtypes to mediate this effect, we analysed the expression of IL-15-mediated phosphorylated STAT5 (pSTAT5) in NK cells that had been pre-activated overnight with either media alone (unprimed) or with each of the selected IFN-I subtypes (**Figure 4A**). Pre-activation with IFNα6 and IFNα14 significantly increased the level of IL-15-induced STAT5-phosphorylation compared to the unprimed controls (**Figure 4B**). In contrast, there was no difference in pSTAT5 expression following pre-activation with IFNα2B and IFNβ, suggesting that not all IFN-I subtypes mediate this priming effect.

### IFNα14 and IFNβ stimulation enhances NK cell anti-leukaemic activity *in vitro* following *ex vivo* expansion

**Figure 5:**
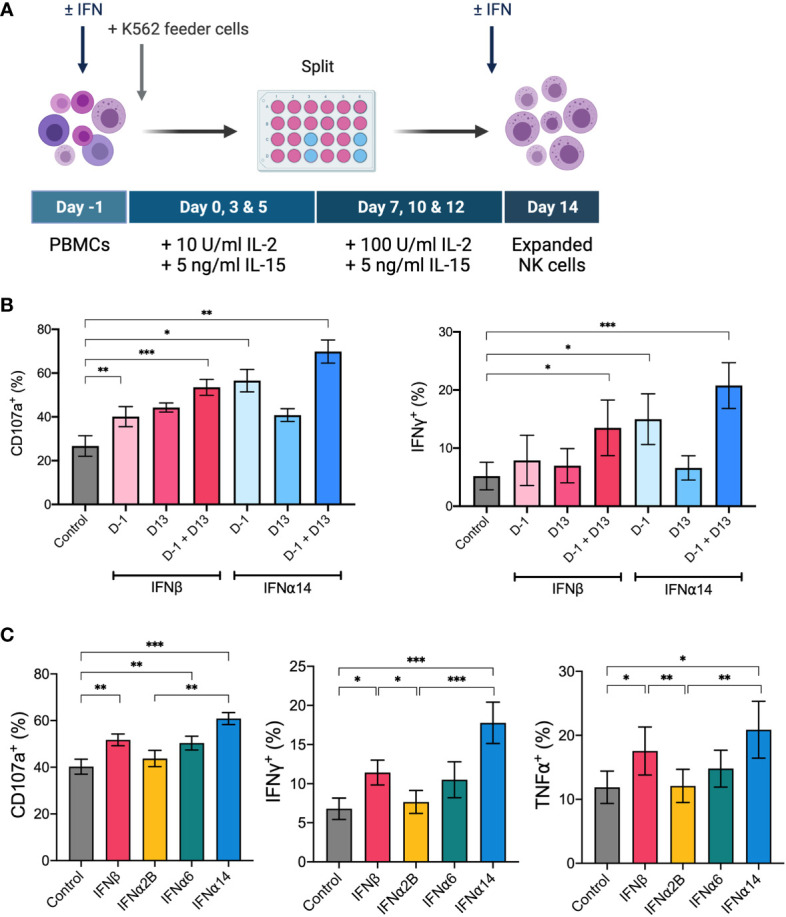
IFNα14 and IFNβ stimulation enhances NK cell activity *in vitro* following *ex vivo* expansion PBMCs were expanded *ex vivo* for 14 days either with or without IFN-I stimulation. **(A)** Schematic of NK cell expansion strategy. On day -1 PBMCs were left unstimulated or stimulated with 100 IU mL^-1^ IFN-I overnight. Following overnight activation, NK cells were expanded for 13 days with IL-15, IL-2, and irradiated K562 feeder cells. On day 13 NK cells were either left unstimulated or stimulated with 100 IU mL^-1^ IFN-I overnight. **(B)** Expanded NK cells that received either no IFN-I activation (control), one dose of IFN-I (D-1 or D13), or two doses of IFN-I (D-1 + D13) were collected on day 14, washed, and assessed for effector function following stimulation with K562 cells for 5 hours at a 2:1 E:T ratio. Summary plots show the mean + SEM percentage of CD56^+^CD3^neg^ NK cells expressing CD107a or producing IFNγ or TNFα. n = 5, 2 independent experiments. Data were compared to the control condition using a paired Student’s *t* test. *p < 0.05, **p < 0.01, ***p < 0.001. **(C)** PBMCs from 16 healthy donors were expanded either without IFN-I stimulation (control) or with IFN-I stimulation on day -1 and day 13. Expanded NK cells were collected on day 14, washed, and assessed for effector function following stimulation with K562 cells at a 2:1 E:T for 5 hours. Summary data show mean ± SEM percentage of CD56^+^CD3^neg^ NK cells expressing CD107a or producing IFNγ or TNFα. Data were compared using a paired Student’s *t* test. *p < 0.05, **p < 0.01, ***p < 0.001.

IL-2 and IL-15 form the backbone of many current *ex vivo* expansion protocols to generate therapeutic NK cells for ACT (27). To investigate the ability for IFN-I stimulation to boost the activity of an NK cell therapy product, we next incorporated the selected IFN-I subtypes into a 14-day *ex vivo* NK cell expansion strategy using IL-2, IL-15 and irradiated K562 feeder cells (**Figure 5A**). We first optimised the timing of IFN-I stimulation using IFNα14 and IFNβ and found that two doses of IFN-I (administered on day -1 and day 13) resulted in the greatest levels of NK cell degranulation and IFNγ production in response to K562 target cells on day 14 (**Figure 5B**). Interestingly, even a single dose of IFN-I given at the start of the culture resulted in significantly increased NK cell activity compared to cells that received no IFN-I. Nevertheless, as the greatest increase in NK cell activity resulted from the two-dose schedule we selected these timepoints to carry forward in this study.

Using this two-dose schedule we next screened each of the selected IFN-I subtypes to identify which subtype(s) generated an NK cell therapy product with heightened anti-leukaemic activity. Upon comparison of NK cell effector functions on day 14, we demonstrated that NK cells activated with each of the selected IFN-I subtypes exhibited varying levels of degranulation and production of IFNγ and TNFα in response to K562 target cells (**Figure 5C**). IFNα2B-activated and control NK cells harboured similar levels of activity, further demonstrating the importance of subtype selection in enhancing NK cell effector function. Notably, NK cells activated with IFNα14 and IFNβ harboured the greatest anti-leukaemic potential, demonstrating significantly greater levels of degranulation and cytokine production compared to both control NK cells and those activated with IFNα2B. It should be noted that activation with IFNα14, but not the other tested IFN-I subtypes, resulted in a small but significantly lower average yield of NK cells on day 14 compared to control (data not shown). Nevertheless, the two highest performing subtypes, IFNα14 and IFNβ, were selected to test the therapeutic efficacy of IFN-I activated NK cells *in vivo*.

### Pre-activation with IFNα14, but not IFNβ, improves efficacy of NK cell ACT against leukaemia

**Figure 6:**
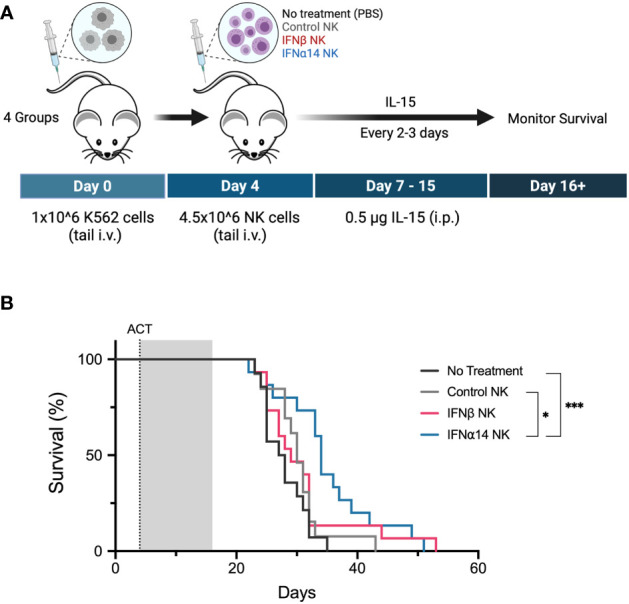
Activation with IFNα14, but not IFNβ, enhances NK cell antitumor responses against K562 leukaemia *in vivo* in NSG mice **(A)** Experimental design. Day 0: NSG mice received 1 x 10^6^ K562 cells *via* tail vein injection. Day 4: NSG mice received 200 µL PBS (no treatment) or 4.5 x 10^6^ 14-day-expanded NK cells (control NK, IFNβ NK, IFNα14 NK) *via* tail vein injection. IL-15 (0.5 µg) was administered on day 0 and every 2-3 days thereafter until day 15. **(B)** Kaplan-Meier curves summarize the survival of mice treated. A total of 13-15 mice were used per group, and each experiment was performed independently three times. Groups were compared using the log-rank (Mantel-Cox) test. *p < 0.05, ***p < 0.001.

Finally, we evaluated the ability of IFNα14- or IFNβ-activated NK cells to control leukaemia *in vivo*. Cohorts of NSG mice were inoculated with K562 leukaemic cells four days prior to receiving adoptive transfer of 4.5x10^6^ activated NK cells which had been expanded either without IFN-I (control NK) or with two doses of IFNα14 or IFNβ (**Figure 6A**). Strikingly, adoptive transfer of IFNα14-activated NK cells significantly prolonged survival compared to both no treatment and treatment with control NK cells (**Figure 6B**). In contrast, no difference in survival was observed following adoptive transfer of IFNβ-activated NK cells. These findings highlight the diverse immunomodulatory effects individual IFN-I subtypes induce and provide further evidence supporting IFNα14 as the optimal subtype for enhancing NK cell functional responses against leukaemia.

## Discussion

NK cells are ideal candidates for cellular therapy due to their intrinsic ability to detect and eliminate leukaemic cells. Although NK cells are unique in their ability to lyse malignant cells without prior sensitisation, cytokines are commonly used to prime NK cells for enhanced antitumor activity. Here, we investigated the ability of each human IFNα subtype and IFNβ to boost NK cell effector functions against leukaemia. We report that individual IFN-I subtypes differ in their capacity to activate NK cell degranulation and cytokine production. Critically, we identified IFNα14 and IFNβ as the most potent subtypes for enhancing NK cell activity *in vitro* following both overnight activation and as part of a prolonged 14-day *ex vivo* activation strategy. When tested therapeutically, adoptive transfer of NK cells activated with IFNα14, but not IFNβ, significantly prolonged survival in an NSG xenograft model of K562 leukaemia. Collectively, these results highlight the differing potencies of IFN-I subtypes as modulators of NK cell immunity and provide support for the development of IFNα14-based NK cellular therapy strategies.

Whilst previous studies have primarily focussed on the antitumour activity of IFNα2, the human genome encodes 12 functional IFNα subtypes and one IFNβ subtype which have been shown to mediate distinct biological effects (7). The differing activities of individual IFN-I subtypes have been attributed to a variety of mechanisms, including binding affinities for IFNAR1 and IFNAR2 subunits (28, 29); stability of the ternary IFN-I/IFNAR complex (30); sensitivity to negative feedback (31); and stimulation of distinct interferon stimulated gene (ISG) expression patterns (29, 32). Interestingly, we report that both IFNα2 variants (IFNα2A and IFNα2B) were among the lowest performing subtypes in our overnight screening assay of NK cell activation. Compared to IFNα2, IFNβ binds to IFNAR with higher affinity (33), forms a longer-lived IFN-I/IFNAR complex (30), and drives a unique gene expression pattern (29), all of which may contribute towards its superior ability to enhance NK cell effector activity. Although IFNα14 has a lower binding affinity to both IFNAR1 (0.68 µM to 3.8 µM) and IFNAR2 (0.7 nM to 1.3 nM) subunits than IFNα2B (34), our findings suggest that both IFNα14 and IFNβ may induce more robust activation of downstream STAT signalling cascades. We observed that stimulation with IFNα14 and IFNβ resulted in greater activation of both STAT1 and STAT3 signalling than IFNα2B. These findings are supported in a recent study by Karakoese and colleagues, in which increased STAT1 and STAT3 signalling was demonstrated in both NK cells and CD4^+^ and CD8^+^ T cells following stimulation with IFNα14 and IFNβ in comparison to IFNα2 (11). Furthermore, both IFNα14 and IFNβ significantly increased the percentage of CD107a^+^ NK and T cells from *in vitro* HIV-infected PBMCs, further demonstrating the enhanced functional potential conferred by these subtypes (11). STAT1 is known to be a critical regulator of NK cell cytotoxicity and IFNγ production (22–24), however there is conflicting evidence about the role STAT3 plays in NK cell function. Although STAT3 activation has been reported to suppress NK cell cytotoxicity in mice (35, 36), there is also evidence for a STAT3-dependent increase in NKG2D expression which may contribute towards enhanced NKG2D-mediated antitumor responses (37, 38). Whilst we observed an increase in NKG2D expression on both IFNα14- and IFNβ-activated NK cells, only IFNα14 stimulation yielded a significant increase in NKG2D expression compared to those activated with IFNα2B. In addition, IFNα14-activated NK cells also demonstrated significantly greater expression of the activating receptor CD160, which has been shown to plan an essential role in IFNγ production (39), and CD108 (semaphorin 7A), a potent immunomodulator that is strongly upregulated on both highly activated and cytokine-induced memory-like NK cells (40), which may have further contributed towards their enhanced effector response. Taken together, these findings highlight the diverse immunomodulatory effects of individual IFN-I subtypes and future studies exploring the mechanisms that drive enhanced anti-leukaemic responses are certainly warranted.

IL-15 currently forms the backbone of many current clinical *ex vivo* expansion protocols to generate NK cells for ACT (27). IL-15 plays an essential role in NK cell development, promoting survival and enhancing NK cell mediated anti-tumour responses (41). In a previous study, IFNα stimulation was reported to prime NK and T cells for greater IL-15-mediated signalling and cytotoxicity (26). Here, we report that our top performing IFN-I subtypes differed in their capacity to prime NK cells for IL-15-mediated signalling, with only IFNα6- and IFNα14-priming resulting in increased pSTAT5 expression following subsequent IL-15 stimulation. As our *ex vivo* expansion protocol utilised IL-15 to generate NK cells for ACT, and mice received recombinant human IL-15 for two weeks following adoptive transfer, this increased priming capacity may contribute towards the superior *in vitro* and *in vivo* antitumour activity displayed by IFNα14-activated NK cells. Indeed, we report that even a single dose of IFNα14 given at the start of the expansion protocol was capable of boosting NK cell effector activity to similar levels obtained from two doses of IFNβ. Furthermore, adoptive transfer of IFNα14-activated, but not IFNβ-activated NK cells, resulted in significantly prolonged survival in our preclinical model of leukaemia. It should also be noted that whilst IL-2 was also present throughout the expansion strategy, IFNα stimulation is not known to prime NK cells for an enhanced response to IL-2 (42). Nevertheless, whilst the extent to which the synergy between IFNα14 and IL-15 contributes towards enhanced antitumour activity remains unclear, further investigation into the biological programs affected by IFNα14 stimulation is required to pinpoint the mechanisms conferring superior anti-leukaemic activity.

In summary, our data provides evidence for diverse IFN-I subtype-specific enhancement of NK cell activity against leukaemia. Although it is now apparent that individual IFN-I subtypes display distinct biological activities, only IFNα2 has been used in the clinic to treat cancer. This study highlights the need for further research into the distinct antitumor and immunomodulatory properties of the remaining IFNα subtypes and IFNβ. Additionally, we describe a strategy whereby NK cells activated with IFNα14 *ex vivo* demonstrate enhanced antitumour activity following adoptive transfer. Identifying the mechanisms underlying the superior activating potential of IFNα14 will provide translational pathways for novel strategies to enhance the efficacy of NK cell-based therapies.

## Data availability statement

The original contributions presented in the study are included in the article/**Supplementary Material**. Further inquiries can be directed to the corresponding authors.

## Ethics statement

The studies involving human participants were reviewed and approved by University of Western Australia Human Research Ethics Committee. The patients/participants provided their written informed consent to participate in this study. The animal study was reviewed and approved by Telethon Kids Institute Animal Ethics Committee.

## Author contributions

SB, BF, and JW designed the experiments. SB, KA, HN, and BF performed the experiments and analysed the data. SB, EJ, BF, and JW wrote the manuscript. SB, KA, HN, SF, EJ, BF, and JW edited the manuscript. SF, EJ, BF, and JW provided supervision. All authors contributed to the article and approved the submitted version.

## Funding

This work was supported by the Australian Government Research Training Program Scholarship at The University of Western Australia and the Lions Cancer Institute Karen & Joshua Chinnery PhD Top Up Scholarship administered by Cancer Council WA (scholarships to SB and HN), the Richard Walter Gibbon Medical Research Scholarship and Rachel Kierath Top-Up Scholarship in Paediatric Cancer Research (scholarships to KA), and funding from the Government of Western Australia Department of Health, Cancer Council WA, Brady Cancer Support Foundation, and the Children’s Leukaemia and Cancer Research Foundation (research funding to BF).

## Acknowledgments

We thank the Telethon Kids Institute Bioresources team for their excellent animal care and the Telethon Kids Institute Flow Facility for assistance.

## Conflict of interest

The authors declare that the research was conducted in the absence of any commercial or financial relationships that could be construed as a potential conflict of interest.

## Publisher’s note

All claims expressed in this article are solely those of the authors and do not necessarily represent those of their affiliated organizations, or those of the publisher, the editors and the reviewers. Any product that may be evaluated in this article, or claim that may be made by its manufacturer, is not guaranteed or endorsed by the publisher.

## Supplementary material

Supplementary Figure 1 IFN-I Activation of NK Cell Function. PBMCs from 50 healthy donors were either left unstimulated or were stimulated with 100 IU mL^-1^ of each IFN⍺ subtype or IFNβ overnight. Following overnight activation, cells were washed and then co-incubated with K562 cells for 5 hours at a 2:1 E:T ratio and NK cell effector functions were assessed. **(A)** Representative gating strategy. NK cell function was assessed as the percentage of CD56^+^CD3^neg^ NK cells expressing CD107a, IFNγ or TNFα, or a combination of two or more of these markers. The percentage of CD107α^+^IFNγ^+^TNFα^+^ NK cells was determined using Boolean gating. **(B)** Percentage of NK cells expressing CD107a, IFNγ or TNFα, or a combination of two or more of these functional markers, following stimulation with selected IFN-I subtypes. Data were compared using paired Student’s *t* tests. **p* < 0.05, ***p* < 0.01, ****p* < 0.001, *****p* < 0.0001.
