# Supplementary figures and images for: Type I interferon subtypes differentially activate the anti-leukaemic function of natural killer cells
Source: Front Immunol. 2022 Nov 24;13:1050718. doi: 10.3389/fimmu.2022.1050718 (PMC9731670; doi:10.3389/fimmu.2022.1050718)

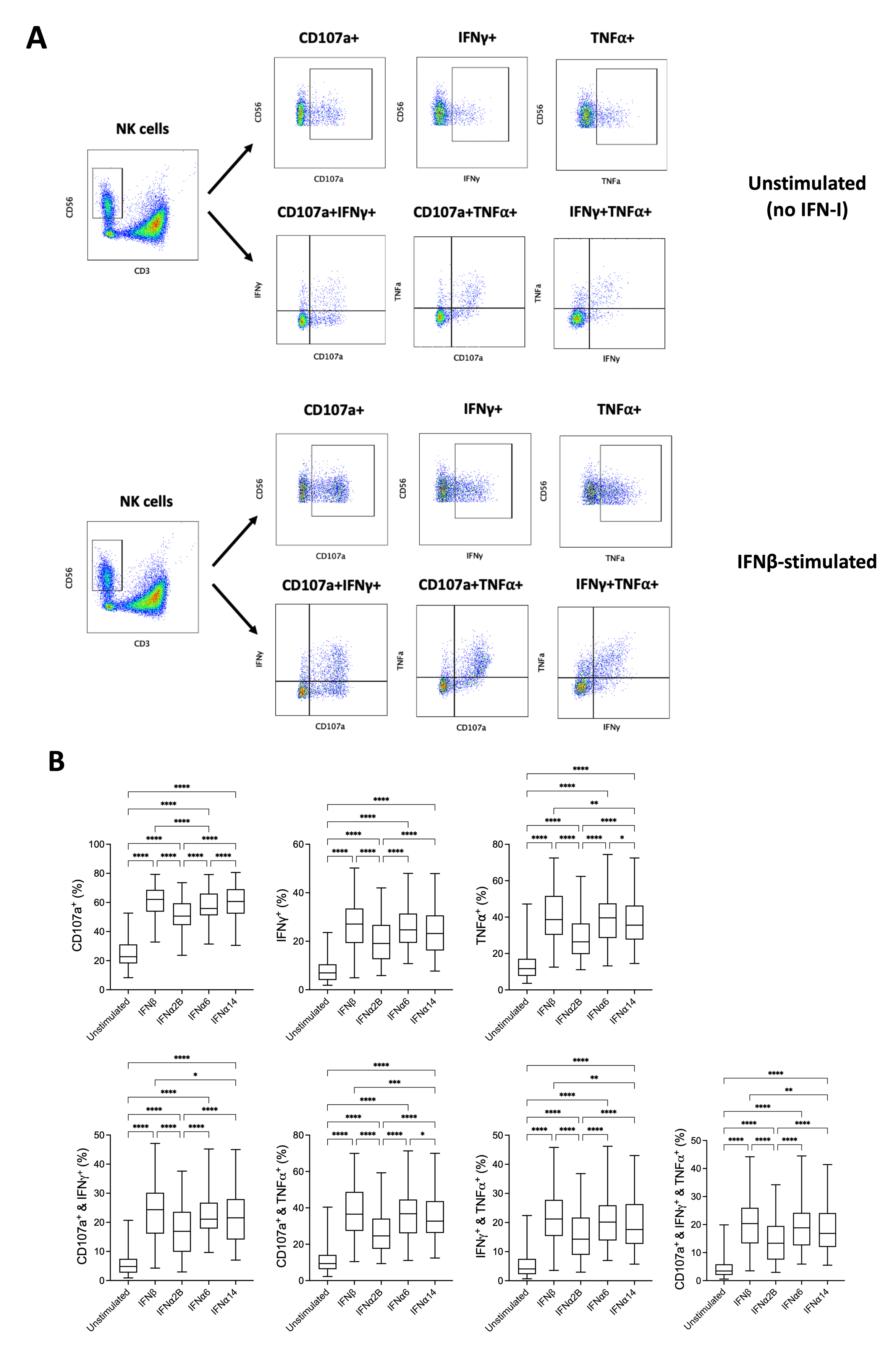

Supplement: Supplementary Figure 1 [file Image_1.tiff]
